# Supplementary material for: Ramadan Fasting during Pregnancy and Health Outcomes in Offspring: A Systematic Review
Source: Nutrients. 2021 Sep 29;13(10):3450. doi: 10.3390/nu13103450 (PMC8540108; doi:10.3390/nu13103450)
Supplement: Supplementary file 1 [file nutrients-13-03450-s001.zip › nutrients-1346713-supplementary.pdf]

Table S1. Data extraction summary table

Reference (year) – setting	Study design	Sample	Quality score	Main findings
Almond and Mazumder (2011) - US, Uganda, Iraq	Retrospective cohort study	US (Michigan): 18 birth cohorts from 1989 – 2006<br>Uganda: census data: 60 birth cohorts<br>Iraq: census data: 20 birth cohorts	4	**Michigan:** Birth weight is lower for Arab pregnancies that overlap with Ramadan (-17.87g, p = 0.03). There are larger effects when Ramadan occurs during the first or second trimester (-20.09, p = 0.05; -25.53g, p = 0.05, respectively). More specifically, if Ramadan coincides with the first two months of pregnancy, the negative effects are larger (-38.0g, p = 0.1; -44.0g, p = 0.05).<br>**Uganda:** Muslims born nine months after Ramadan have a statistically significant increase in the likelihood of a disability of any kind (0.819%, p = 0.02). Mean disability rate is 3.8%, thus the effect is substantial at 22% (0.819/3.8). More specifically, the increased incidence of a mental or learning disability (0.250%, p = 0.001). Mean rate of 0.14%, thus Ramadan early in pregnancy nearly doubles likelihood of a disability related to mental or learning disability.<br>**Iraq:** Full exposure to Ramadan nine months before birth is associated with a 0.33 percentage point increase in the probability of having a disability (p = 0.016).
Almond, Mazumder and van Ewijk (2014) - England	Retrospective cohort study	**Population:** students who were assessed at Key Stage 1 between 1998 and 2007 selected from student register (treatment group: Pakistani/Bangladeshi, control group: Caribbean)<br>**N** = 4.6 million (including British control)	6	**Reading, writing and mathematics test scores are 0.05-0.08 standard deviations lower for students exposed to Ramadan in the first three months of pregnancy (p < 0.05).** There were no statistically or quantitatively significant negative effects of prenatal Ramadan exposure on test scores for Caribbean students (control).
Altunkeser & Körez (2016) - Turkey	Case-control	**Population:** fasting or non-fasting women in their second or third trimester<br>**N =** 119 (exposed: N = 61, unexposed: N = 58. Group 1a: 1-10 days, Group 1b: 11-20 days, Group 1c: 21-30 days	6	**Fasting did not influence amniotic fluid amount as long as absolute food and fluid intake was sufficient.**<br>Low AF (amniotic index <5 cm) - exposed: 8.2%, non-exposed: 5.2%.<br>AI – exposed: 13.02±1.81, non-exposed: 13.03±2.35<br>Delivery week – exposed: 39.25, non-exposed: 38.8 (p = 0.18)<br>Birth weight – exposed: 3305.74±434.47, non-exposed: 3268.55±654.21 (p = 0.366)<br>Delivery (caesarean/natural) – exposed: 15/46, 2 non-exposed: 25/31 (p = 0.022)

Alwasel et al. (2010) – Saudi Arabia	Retrospective cohort study	**Population:** Birth records of singleton babies born to Saudi nationals in King Saudi Hospital (Unizah) in a four-year period (August 2000 – April 2004)<br>**N** = 7083 (outside of pregnancy: 1822, first trimester: 1598, second trimester: 1934, third trimester: 1729)	4	**The birth weight of babies exposed in utero (first, second, and third trimester) to Ramadan did not significantly differ from those not exposed, in both girls and boys.**<br>Birth weight (girls) – outside of pregnancy: 3.17, first trimester: 3.20 ($p = 0.3$), second trimester: 3.18 ($p = 0.7$), third trimester: 3.20 ($p = 0.2$)<br>Birth weight (boys) – outside of pregnancy: 3.28, first trimester: 3.31 ($p = 0.2$), second trimester: 3.29 ($p = 0.7$), third trimester: 3.30 ($p = 0.3$)
Alwasel et al. (2011) – Saudi Arabia	Retrospective cohort study	**Population:** Singleton babies born to Saudi nationals at term in 2007. From each month's births, first 50 boys and first 50 girls who met inclusion criteria were selected<br>**N** = 967 babies (661 babies had maternal data for relevant analysis – not in utero: 163, first trimester: 154, second trimester: 178, third trimester: 166)	5	**Babies whose mothers were exposed in utero in the second trimester to Ramadan grew differently. Boys were longer. Girls had a shorter gestation period. The brain growth of boys was better developed than that of girls.**<br>Length (boys) – not in utero: 51.1cm, first trimester: 52.1cm ($p = 0.07$), second trimester 52.3cm ($p = 0.005$), third trimester: 51.7cm ($p = 0.8$)<br>Gestation (girls) – not in utero: 39.8 weeks, first trimester: 39.8 weeks ($p = 0.9$), second trimester: 39.4 weeks ($p = 0.04$), third trimester: 39.8 weeks ($p = 0.9$)<br>Babies (boys and girls) whose mothers were in utero during Ramadan, did not differ significantly in birth weight, head circumference, or chest circumference.
Alwasel et al. (2013) – Tunisia	Retrospective cohort study	**Population:** Singleton babies born at term during May 2011 to April 2012 in the Gafsa Hospital<br>**N** = 1321 (not in utero: 287, T1: 336, T2: 355, T3: 343)	4	**Babies whose mothers were in utero during Ramadan were smaller, lighter and had a shorter HC.**<br>Birth weight – not in utero: 3469, any trimester: 3373 ($p = 0.003$), first trimester: 3360 ($p = 0.006$), second trimester: 3363 ($p = 0.005$), third trimester: 3395 ($p = 0.06$)<br>HC – not in utero: 35.3, any trimester: 35.2 ($p = 0.05$), first trimester: 35.2 ($p = 0.1$), second trimester: 35.1 ($p = 0.04$), third trimester: 35.2 ($p = 0.2$)<br>Length – not in utero: 50.3, any trimester: 50.2 ($p = 0.1$), first trimester: 50.2 ($p = 0.2$), second trimester: 50.1 ($p = 0.06$), third trimester: 50.2 ($p = 0.4$)

Arab and Nasrollahi (2001) – Iran	Cross-sectional study	**Population:** full term healthy pregnant women delivering singletons at term in Hamadan City Hospital or delivery facility centers just after the month of Ramadan in 1999 and up to 9 months thereafter<br>**N** = 4343 (days fasted – non-fasted: 1257, fasted 1-9 days A: 752, fasted 10-19 days B: 590, fasted more than 20 days C: 1744; trimesters – non-fasted: 1257, T1: 957, T2: 1207, T3: 922)	4	**No interrelationship of fasting and birth weight in any trimester. Lowest mean birth weight in women who fasted 1-9 days and the highest in women who fasted 20 days or more. The difference was significant.**<br>**Frequency of low birth weight (LBW) is increased in women who fasted during their second trimester, although not statistically significant.**<br>Birth weight – non-fasted: 3198±486.7g, A: 3168±513.5g, B: 3137±522.6g, C: 3142.0±492.8g (A-C p = 0.005; non-fasting-C p = 0.002)<br>LBW – non-fasting: 6.5%, A: 7.8%, B: 6.1%, C: 5.5% (p = NS)<br>LBW – non-fasting: 6.5%, first trimester: 4.9%, second trimester: 7.5%, third trimester: 5.9% (p = 0.09, although worthy of attention)
Awwad et al. (2012) – Lebanon	Prospective cohort study	**Population:** Women with singleton pregnancies presenting for prenatal care (20-34 weeks of gestation) during the month of Ramadan (September 2008)<br>**N** = 402 (control group:201, fasted group: 201)	8	**Proportion of women who had PTD was comparable between fasted and non-fasted groups. No difference in occurrence of PTD whether women fasted during first, second, or third trimester.**<br>**Mean birth weight was found to be lower in the fasted group, as well as caesarean delivery rate.**<br>PTD (<37 weeks) – control group: 10.4%, fasted group: 10.4% (p = 1.000)<br>PTD (<32 weeks) – control group: 0.5%, fasted group: 1.5% (p = 0.623)<br>Birth weight – control group: 3202±473g, fasted group: 3094±467 (p = 0.024)<br>Caesarean delivery – control group: 39.3%, fasted group: 28.4% (p = 0.027)
Azizi (2004) – Iran	Retrospective cohort study	**Population**: children aged 4 to 13 years were selected from 15 schools via a questionnaire filled out by mothers<br>**N** = 191 (controls: 93, fasted: 98)	6	**No significant differences were observed between the IQ scores.**<br>Full-scale IQ, performance IQ, verbal IQ of the two groups – case: 111±10, 109±11, and 110±11, control: 112±10, 110±11, 110±11; p = NS, respectively).<br>SES accounted for 17% of variance in IQ scores.

Bayoglu Tekin et al. (2018) - Turkey	Cross-sectional study	**Population:** Pregnant women who were in the third trimester recruited at a University Hospital antenatal outpatient clinic.<br>**N =** 48 (exposed: 23, non-exposed: 25).	2	**Blood urea nitrogen, potassium and hematocrit levels were significantly higher in fasting women than non-fasting women.**<br>Fetal weight - exposed: 3457.1±348.5, non-exposed: 3312.5 ±666.4 (p = 0.58)<br>Maternal weight – exposed: 75.0±14, non-exposed: 77.9±12.2 (p = 0.59)<br><br>**Doppler indices increased in women before the Ramadan as compared to after, from which some of them were statistically significant.**<br>UA S/D – exposed: 2.7±0.3, unexposed: 2.7±0.8; (p = 0.98<br>fRA PI – exposed: 2.2±0.2, unexposed: 1.9±0.4; p = 0.02<br>fRA RI – exposed: 0.09±0.1, unexposed: 0.7±0.1; p = 0.04<br>fRA S/D – exposed: 8.8±2.2, unexposed: 6.7±3.9; p =0.15
Boskabadi, Mehdizadeh and Alboumiri (2014) – Iran	Cross-sectional study	**Population:** Non-random sampling applied at Qaem Hospital (2010- 2014) to select healthy, fasting women.<br>**N** = 641 (fasted ≤10 days A: 137, fasted 11-20 days B: 160, fasted 21-30 days C: 344)	4	**Mean head circumference at birth was smaller and birth weight was lighter when mother fasted during first trimester. These differences were statistically significant.**<br>Incidence of prematurity and PTD delivery was lower in pregnant women who fasted for longer periods, although not statistically significant.<br>Prematurity – A: 15.7%, B: 7.6%, C: 6.9% (p = 0.09)<br>Head circumference at birth – A: 34±2cm, B: 35±1.13cm, C: 35±1cm (p = 0.007)<br>Height at birth – A: 50±2cm, B: 50±2cm, C: 50±3cm (p = 0.113)<br>Weight at birth – A: 3100±83g, B: 3300±75g, C: 3300±80g (p < 0.001)<br>Fetal weight - exposed: 3457.1±348.5, non-exposed: 3312.5 ±666.4 (p = 0.58)<br>Maternal weight – exposed: 75.0±14, non-exposed: 77.9±12.2 (p = 0.59)
Cross, Eminson and Wharton (1990) – England	Retrospective cohort study	**Population:** Birth records of babies born at full term drawn from Birmingham births data from 1964 to 1984.<br>**N** = 31808 (Asian Moslem: 13351, white: 13351, non-Moslem Asian: 5106)	3	**Ramadan had no effect on mean birth weight at any stage of pregnancy in cumulative and quinquennial analysis.**<br>There was an increase in low birth weight (4.5% to 8.0%) among babies who were born at full term when Ramadan had occurred during the second trimester, although not significant (see Figure 3).

Daley et al. (2017) – England	Prospective cohort study	**Population:** pregnant women who attended the Bradford Royal Infirmary at 26-28 weeks gestation between March 2007 and December 2010<br>**N** = 5,156 (exposed: N=479, unexposed: N=4,677)	5	**LBW: No significant difference found in birthweight and term of delivery of the ratio of exposed infants versus non-exposed infants.**<br>LBW - exposed: 9.6%, -non-exposed: 8.9% (OR 1.12)<br>PTD - exposed: 5.8%, -non-exposed: 4.8% (OR 1.21)<br>Birth weight: 24.3g difference
Dikensoy et al. (2008) – Turkey	Prospective cohort study	**Population:** Consecutive healthy women with singleton uncomplicated pregnancies of ≥20 weeks of gestation were enrolled if consented.<br>**N** = 65 (fasted: 36, non-fasted: 29)	7	**Fasting during Ramadan had no significant adverse effect on intrauterine fetal development or the fetus' health (fetal BPD, FL, EFBW, fetal BPP, AFI, etc.).**<br>Increase in fetal BPD – fasted: 4.5mm, non-fasted: 4.0mm (p = 0.621)<br>Increase in fetal FL – fasted: 3.2mm, non-fasted: 3.0 (p = 0.542)<br>Increase in EFBW – fasted: 221g, non-fasted: 214g (p = 2.19)<br>AFI – fasted: 12.9mm, non-fasted: 13.3mm (p = 434) BPP – fasted: 7.0, non-fasted: 7.8 (p = 0.326)
Hizli et al. (2012) – Turkey	Prospective cohort study	**Population:** Healthy women with singleton uncomplicated pregnancies (August 11 2010 – September 9 2010)<br>**N** = 110 (fasted: 56, control: 54)	7	**Fasting of healthy women during pregnancy has no adverse effect on fetal AFI, birth weight, gestation age at delivery, and caesarean section rate.**<br>AFI – fasted: 130.5±36.8, non-fasted: 125.1±27.0 (p = 0.07)<br>Birth weight – fasted: 3404±400g, non-fasted: 3285±351g (p = 0.1)<br>Gestational age at delivery – fasted: 39.3 weeks, non-fasted: 38.9 weeks (p = 0.08)<br>Caesarean delivery – fasted: 39.3%, non-fasted: 37.0% (p = 0.9)
Jamilian et al. (2015) – Iran	Prospective cohort study	**Population:** healthy pregnant women at gestational age of ≥ 28 weeks recruited from Taleghani Hospital<br>**N** = 317 (fasted 1-9 days A: 83, fasted 10-19 days B: 13, fasted 20-30 days C: 18, control group: 203)	7	**Ramadan fasting (regardless of number of days) has no effect on outcome of pregnancy and fetal health.**<br>Birth weight – control group: 3104±371g, A: 3168±677g, B: 3195±391g, C: 3331±442g (p = 0.6)<br>Delivery (caesarean/natural) – control group: 21/77, A: 23/19, B: 1/8, C: 0/9 (p = 0.08)<br>Length neonate – control group: 50.4±2.3cm, A: 50.6±2.7cm, B: 51.0±1.1cm, C: 50.8±2.7cm (p = 0.7)<br>Head circumference baby – control group: 34.8±1.0cm, A: 35.7±1.7cm, B: 35.4±0.8cm, C: 35.3±1.2cm (p = 0.5)

Karateke et al. (2015) – Turkey	Prospective cohort study	**Population:** Healthy pregnant women were followed for fetal outcomes from June 2014 until January 2015<br>**N** = 240 (Trimester 1 – fasted: 40, non-fasted: 40; Trimester 2 – fasted: 40, non-fasted: 40; Trimester 3 – fasted: 40, non-fasted: 40)	6	**There were no significant differences between the two groups in terms of increases on fetal BPD, EFBW, newborn weight, delivery and LBW when prenatally exposed during any trimester.**<br>**A statistically significant increase was observed in AFI in second trimester.**<br><u>Trimester 1</u><br>Increase of fetal BPD – fasted: 3.4mm, non-fasted: 3.6mm (p = NS)<br>Newborn weight – fasted: 3015±615.3, non-fasted: 3052±595.6 (p = NS)<br>LBW – fasted: 5%, non-fasted: 2.5% (p = NS)<br>Delivery (normal/C) – fasted: 28/12, non-fasted: 30/10 (p = NS)<br><br><u>Trimester 2</u><br>Increase of fetal BPD – fasted: 3.7mm, non-fasted: 3.9mm (p = NS)<br>Increase of fetal FL – fasted: 3.4mm, non-fasted: 3.6mm (p = NS)<br>Increase of EFBW – fasted: 370g, non-fasted: 390g (p = NS)<br>AFI – fasted: 11.4mm, non-fasted: 16.2mm (p = 0.02)<br>Newborn weight – fasted: 2925±542.4g, non-fasted: 3013±524.2 (p = NS)<br>LBW – fasted: 2.5%, non-fasted: 2.5% (p = NS)<br>Delivery (normal/C) – fasted: 34/6, non-fasted: 32/8 (p = NS)<br><br><u>Trimester 3</u><br>Increase of fetal BPD – fasted: 3.9mm, non-fasted: 4.1mm (p = NS)<br>Increase of fetal FL – fasted: 3.6mm, non-fasted: 3.7mm (p = NS)<br>Increase of EFBW – fasted: 650g, non-fasted: 670g (p = NS)<br>AFI – fasted: 10.8mm, non-fasted: 12.4mm (p = NS)<br>Newborn weight – fasted: 3029±530.5g, non-fasted: 3045±572.6 (p = NS)<br>LBW – fasted: 2.5%, non-fasted: 2.5% (p = NS)<br>Delivery (normal/C) – fasted: 29/11, non-fasted: 31/9 (p = NS)

Karimi and Basu (2018) – 37 countries	Retrospective cohort study	**Population:** data from 98 demographic and health surveys (DHS) in 37 countries that have at least a 10% Muslim population.<br>**N** = 308,879 singleton children.	4	**Negative associations were found between exposure to Ramadan and Muslim male children's height at ages 3 and 4 years.**<br>- Height-for-age Z-score for males age 4 (Muslim vs non)<br>Full 30-day exposure in trimester 1: -0.098SD ($p < 0.01$)<br>Full 30-day exposure in trimester 2: -0.107SD ($p < 0.05$)<br>Full 30-day exposure in trimester 3: -0.025SD ($p > 0.1$)<br>- Height in millimeters of a full 30-day exposure for males (Muslim vs non)<br>Age 0: 0.1 (95%CI -1.7, 1.8)<br>Age 1: 0.1 (95%CI -1.6, 1.8)<br>Age 2: -0.6 (95%CI -2.9, 1.6)<br>Age 3: -1.0 (95%CI -4.4, 2.4)<br>Age 4: -3.2 (95%CI -5.0, -1.4)<br>Effects tend to be stronger in West Africa and Central Asia, as well as in more religious countries.<br>Females' children's height is not negatively associated with prenatal exposure to Ramadan.
Kavehmanesh and Abolghasemi (2004) – Iran	Retrospective cohort study	**Population**: Healthy women with no history of significant illnesses who gave birth at the Najmieh and Baqiyatallah hospitals from January 1 to end of September 2000<br>**N** = 539 (fasted: 284, non-fasted: 255)	5	**On the univariate analysis, neonatal birth weight of the fasted group was 100g more than those of the non-fasted group ($p = 0.009$), although 71 g (ß, SE(ß): 43.4) heavier in multivariable analysis ($p = 0.1$).**<br>PTD – fasted: 2.8%, non-fasted: 3.9% ($p = 0.47$)<br>Birth weight – fasted: 3265±444, non-fasted: 3165±440 ($p = 0.009$)

Kunto and Mandemakers (2018) - Indonesia	Retrospective cohort study	**Population:** data from 5 waves of the Indonesian Family Life Survey, in the period 1993–2015.<br>**N =** 45,246 observations of 21,723 children born to 9771 mothers,	5	**Although no main-effects of prenatal exposure to Ramadan on stature could be found, results suggest that prenatal exposure to Ramadan negatively affects stature when age (0-4 y, 5-9, 10-14 and 15-19) and timing (1st, 2nd, 3rd trimester) are considered.**<br>Some main results for the religious groups:<br>- A lag in height growth for children exposed to Ramadan starts in late childhood (5-9y) and becomes significant in late adolescence (15-19y). Effects become more evident when timing is considered. E.g.: HAZ difference 15-19y, independent of timing: -0.077 SD ($p < 0.10$) HAZ difference 15-19y, exposed in 1st trimester: -0.105 SD ($p < 0.05$)<br>- A similar pattern for children exposed to Ramadan was found for BMI, although not significant, e.g.:BMI difference 10-14y, exposed in 3rd trimester; -0.09 SD ($p < 0.10$)<br>Surprisingly, positive effects on stature were found for exposed less-religious Muslim children (their mothers prayed < 5 times a day) that peak in early adolescence (10– 14y) and negative effects on stature for exposed non-Muslim children that occur only in early childhood (0 – 4y) were found. This could be explained by health and socioeconomic factors.

Majid (2015) – Indonesia	Retrospective cohort study	**Population:** sampling children aged 7-15 in 2007 from Wave 4 of the Indonesian Family Life Survey<br>**N** = 3901 (Muslims: 3512, Non-Muslims: 380)	6	**Children potentially exposed score 7.4% lower in their cognitive scores and 8.4% lower in math scores. Largest effects were observed in the first trimester.**<br><u>Muslims vs. Non-Muslims</u><br>Muslims – cognitive scores: -0.074 (p < 0.001); math scores: -0.084 (p < 0.001); total scores: -0.082 (p < 0.001)<br>Non-Muslims – cognitive scores: 0.083 (p = NS); math scores: 0.036 (p = NS); total scores: -0.062 (p = NS)<br><br><u>Trimesters</u><br>Exposed T1 – cognitive scores: -0.074 (p < 0.1); math scores: -0.065 (p = NS); total scores: -0.078 (p < 0.05)<br>Exposed T2 – cognitive scores: -0.014 (p = NS); math scores: -0.034 (p = NS); total scores: -0.024 (p = NS)<br>Exposed T3 – cognitive scores: -0.051 (p = NS); math scores: -0.069 (p < 0.1); total scores: -0.055 (p < 0.1)
Makvandi, Nematy and Karimi (2013) – Iran	Cross-sectional study	**Population:** healthy pregnant women within the age range of 18-35 who were hospitalized and had delivered their babies in the Sina Academic Hospital in 2013<br>**N** = 300 (fasted: 150, non-fasted: 150)	4	**No significant difference between fasted and non-fasted groups in birth weight, head circumference, height and prevalence of low birth weight when exposed to Ramadan in the <u>third trimester</u>.**<br>Birth weight – fasted: 3172.01±538.86g, non-fasted: 3173.91±501.55g (p = 0.97)<br>Head circumference – fasted: 34.70±1.04cm, non-fasted: 33.91±1.30cm (p = 0.09)<br>Height – fasted: 49.40±2.60cm, non-fasted: 51.08±2.04cm (p = 0.12)<br>LBW – fasted: 5.33%, non-fasted: 4% (p = 0.33)
Mirghani et al. (2002) – UAE	Cross-sectional study	**Population:** Between November 17 2001 and December 12, 2001, a consecutive sample of healthy pregnant women observing Ramadan was recruited from Al-Ain Hospital<br>**N** = 162 (fasted: 81, non-fasted: 81)	4	**No effect of Ramadan on the estimated fetal weight during pregnancy.**<br>Fetal breathing movements in fasting pregnant women significantly reduced compared to non-fasted group. All other biophysical profile components were normal in the two groups.<br>**A significant difference between the two groups was noted in biophysical score.**<br>Estimated fetal weight – fasted: 2488±599g, non-fasted: 2362±643g (p = NS)<br>Breathing movements – fasted: 63%, non-fasted: 87% (p = 0.001)<br>Gross body movements – fasted: 100%, non-fasted: 100% (p = NS)<br>Fetal tone – fasting: 100%, non-fasted: 100% (p = NS)<br>Normal amniotic fluid volume – fasted: 100%, non-fasted: 98% (p = NS)<br>Biophysical score of 6/8 – fasted: 37%, non-fasted: 13.6% (p = 0.001)

Moradi (2011) – Iran	Case-control study	**Population:** Healthy pregnant women of T2 or T3 with singleton pregnancy at Shahid Beheshti Hospital (21 August – 23 September 2009)<br>**N** = 52 (fasted: 25, non-fasted: 27)	5	**Increases in growth indices (BPD, FL, AC, EFW) were similar in fasted and non-fasting groups. No undesirable effect on fetal growth due to Ramadan fasting of mothers in second and third trimester.**<br>ΔBPD – fasted: 11.64±3.6mm, non-fasted: 11.81±3.6mm (p = 0.86)<br>ΔFL – fasted: 12.4±8.8mm, non-fasted: 10.0±3.3mm (p = 0.19)<br>ΔAC – fasted: 42.1±13.0mm, non-fasted: 46.2±13.6 mm (p = 0.27)<br>FWG – fasted: 571±339g, non-fasted: 562±301g (p = 0.92)
Ozturk et al. (2011) – Turkey	Prospective controlled study	**Population:** healthy women with singleton pregnancies attending the Obstetrics and Gynecology Department of Gaziantep University from 1-29 September 2008 in T2<br>**N** = 72 (fasted: 42, non-fasted: 30)	6	**Maternal fasting during Ramadan in the second trimester does not have a significant effect on birth weight or preterm delivery.**<br>Birth weight – fasted: 3310g, non-fasted: 3518g (p = 0.45)<br>PTD – fasted: 1 case, non-fasted: 1 case (p = NS)
Petherick, Tuffnell and Wright (2014) – England	Prospective cohort study	**Population:** Asian or Asian-British Muslim women who had lived singleton births with a linked maternity record<br>**N** = 310 (fasted: 128, non- fasted: 172 → trimester 1: 42, trimester 2: 74, trimester 3: 11)	9	**No association observed between fasting and PTD, low birth weight, birth weight either before or after adjustment for other covariables.**<br>Birth weight – non-fasted: 3133g, fasted: 3219.3g (OR 75.07 p = 0.62)<br>Gestational length – non-fasted: 39.1 weeks, fasted: 39.2 weeks<br>PTD – non-fasted: 3.5%, fasted: 6.3% (OR 0.72 p = 0.71)<br>LBW – non-fasted: 8.1%, fasted: 6.3% (OR 1.82 p = 0.25)<br>Relationship with duration on fasting (days) is not significant.
Pradella and van Ewijk (2018)	Retrospective cohort study	**Population:** data from the Indonesian Family Life Survey (waves 2 – 4), between 1997 and 2008.<br>**N =** 28,489 (26,313 Muslims, 2,176 non-Muslims)	4	**Breathing difficulties and wheezing prevalence was higher among adult Muslims who had been in utero during Ramadan, independent of the pregnancy phase in which the exposure to Ramadan occurred. Significant associations were found only among males.**<br>Exposed versus non exposed Muslims:<br>Any breathing difficulty: OR = 1.17 (95% CI 1.02, 1.35; p = 0.022)<br>Females: OR = 1.14 (95% CI 0.94, 1.39; p = 0.176)<br>Males: OR = 1.21 (95% CI 0.99, 1.47; p = 0.061)<br>Wheezing: OR = 1.26 (95% CI 0.97, 1.30; p = 0.087)<br>Females: OR = 1.09 (95% CI 0.75, 1.57; p = 0.657)

				Males: OR = 1.45 (95%CI 1.00, 2.12; p = 0.051)<br>Associations were most pronounced for smokers and the risk of experiencing wheezing after Ramadan exposure increased with age.
Rezk et al. (2016) - Egypt	Prospective case-control study	**Population:** pregnant women of T3 with singleton pregnancy at the department of Obstetrics and Gynecology at Menoufia University Hospital Menoufia, June 17 – July 16 2015<br>**N** = 450 (fasted: 210, non-fasted:240)	8	**There were no significant differences in fetal well-being parameters or neonatal outcomes found between the case and control group:**<br>AFI – exposed: 8.9±1.1, non-exposed: 9±1.2, p > 0.05<br>Birth weight – exposed: 3.49±0.2kg, non-exposed: 3.52±0.3, p > 0.05<br>Caesarean delivery - exposed: 58 (27.7%), non-exposed: 68(28.4%)<br>Gestational age at delivery – exposed: 40.0±0.78, non-exposed: 39.9±0.8, (p> 0.05<br>C/U ratio – exposed: 1.46±0.21, non-exposed: 1.48±0.23, p > 0.05<br>In this study, fasting was not found to affect biophysical profile, Amniotic Fluid Index (AFI), the reactivity of Non-Stress Test (NST) and Doppler indices of the umbilical and middle cerebral arteries (MCA) (p > 0.05).
Safari, Piro and Ahmad (2019) - Iraq	Case-control study	**Population:** healthy pregnant Muslim women aged 18–35 = with live singleton birth. Data collected from October 2017 to January 2018 in Hawler Maternity Teaching Hospital of Erbil, Iraq.<br>**N = 301** (155 fasting during second trimester, 146 non-fasting)	5	**No association was found between fasting (yes/ no and duration: 1-10 days, 11-20 days, > 20 days) and preterm labour, preeclampsia, mode of delivery, low birth weight, fetal weight, height and head circumference at birth, and fetal Apgar score.**
Sakar et al. (2015) – Turkey	Prospective case-control study	**Population:** singleton pregnancies at second or third trimester<br>**N** = 106	5	**A comparison was made between fasting and non-fasting women: at the end of the Ramadan the differences in increase in biparietal diameter, head circumference, and femur length was significant (P < 0.05).**<br>Δ biparietal diameter (mm), fasting: 9.69 ± 3.07, non-fasting: 10.74 ± 1.99, p = 0.041<br>Δ head circumference (mm), fasting: 35.12 ± 12.22, non-fasting: 39.12 ± 7.42, p = 0.046<br>Δ femur length (mm), fasting: 8.57 ± 2.56, non-fasting: 9.98 ± 1.86, p = 0.002<br><br>Increase in fetal weight (fetal weight gain) and uterine and umbilical artery Doppler indices (resistance index (RI), pulsatility index (PI), and systolic to diastolic (S/D) ratio) were similar in the two groups.

				No statistical differences were found when comparing AFI between groups. When the groups were evaluated separately, an increase of AFI was significant in the non-fasting group ($P < 0.05$).
Sakar et al. (2016) – Turkey	Prospective case-control study	**Population:** women at or after 37 weeks of gestation<br>**N =** 338 (168 fasting; 170 non-fasting)	7	**No significant differences were found in birth indices (HC, birth weight, height). However, there was a significantly heavier weight of the placenta measured in exposed versus non-exposed women.**<br>HC – exposed: 34.54±1.08, -non-exposed: 34.62±0.88, p = 0.430<br>Birth weight – exposed: 3230.5±500.62, non-exposed: 3242±382.87, p = 0.801<br>Placental weight: 633.60±143.60, non-exposed: 601.97±133.15, p = 0.037<br>Height – exposed: 50.12±1.41, non-exposed: 50.05±1.03, p = 0.624
Sarafraz et al. (2014) – Iran	Retrospective cohort study	**Population:** Women within the age range of 20-35 years who delivered a child in one of two hospitals in Kashan, and fasted during one of the pregnancy trimesters<br>**N** = 293	6	**There were no significant associations between birth weight and number of fasting days.**<br>The mean birth weights of the fasted group were lower than the non-fasted group, although it was not statistically significant.<br><br><u>Birth weight</u><br>T1 – fasted: 3360.133±481.603, non-fasted: 3378.484±350.215<br>T2 – fasted: 2976.923±698.991, non-fasted: 3050.000±241.522<br>T3 – fasted: 3250.000±212.132, non-fasted: 3400.000±102.122<br><br>Birth weight – non-fasted: 3348.461±339.949, fasted 1-10 days: 3198.285±584.5686, fasted 11-20 days: 3429.206±462.6372, more than 20 days: 3326.346±483.4729 (p = 0.145)
Sarafraz et al. (2015) – Iran	Retrospective cohort study	**Population:** Women who fasted at least one day during Ramadan and who were admitted for delivery in one of two public hospitals in Kashan<br>**N** = 250 (first trimester: 112, second trimester: 68, third trimester: 70)	7	**Differences in means of birth weight were statistically significant. Frequency of low birth weight was not statistically significant.**<br>Birth weight – first trimester: 3411±529.88g, second trimester: 3214±463.56g, third trimester: 3336±444.89g (p = 0.03)<br>LBW – first trimester: 8.9%, second trimester: 8.8%, third trimester: 7.1% (p = 0.9)

Savitri et al. (2014) – Netherlands	Prospective cohort study	**Population:** Muslim women who were pregnant during the month of Ramadan in 2010 with a gestational age over 4 weeks and did not give birth during Ramadan<br>**N** = 130 (non-fasted: 60, fasted half a month or less: 21, fasted more than half a month: 49)	7	**Newborns exposed to Ramadan had a reduced birth weight compared to those not exposed (-198g, 95% CI -447, 51; p = 0.12). There were no differences in birth weight if fasting was in T2/T3.**<br>Women who fasted more than half a month had newborns of lower birth weight than those who did not fast. Women who fasted less than half a month had newborns of heavier birth weight. These associations were not statistically significant.<br><br>Model 4 (adjustment for maternal smoking status and ethnicity, parity, pregnancy risk classification and SES)<br>Fasted in T1: -272.1 (95% CI -547, 3; p = 0.05)<br>Fasted in T2: 28.3 (95% CI -235, 292; p = 0.83)<br>Fasted in T3: 46.8 (95% CI 289, 383; p = 0.78)
Savitri et al. (2018) – Indonesia	Prospective cohort study	**Population:** pregnant women in Budi Kemuliaan Hospital, Jakarta from July 2012 until October 2014<br>**N =** 139 (fasted: 110, non-fasted: 29)	7	**There was no significant difference in birth weight between infants exposed to fasting versus non-fasting.**<br>Model 3 Birth weight regression coefficients (adjusted for smoking, family income, maternal education, GMI, gestational duration and parity)<br>Fasted in **T2**: -0.7 (95% CI-260.8, 259.4)<br>Fasted in **T3**: -38.1 (95% CI-244.6, 168.3)<br>Fasted in **T3**: -144.1 (95% CI-360.2, 72.0)<br>Birth weight – exposed: 3107g, unexposed: 3022g
Savitri et al. (2019) - Netherlands	Retrospective cohort study	**Population:** all births between 2000 and 2010 of mothers recorded as Mediterranean or ethnically Dutch, in the Perinatal Registry of the Netherlands (Perined)<br>**N** = 1,620,757 children (139,322 Mediterranean; 1,481,435 ethnically Dutch)	4	**The occurrence of a Ramadan during pregnancy (in trimester 1, 2, 3 or any trimester) among Muslims was not associated with altered birth weight, gestational length, newborn's sex, perinatal mortality, low Apgar, or mild congenital anomalies.**<br>Severe congenital anomalies occurred more frequently after Ramadan exposure (OR: 1.17; 95% CI: 1.00, 1.37), but association became non-significant when adjusting for multiple testing.

Schoeps et al. (2018) – Burkina faso	Retrospective cohort study	**Population**: data from Nouna Health and Demographic Surveillance System (HDSS) in northwestern Burkina Faso. All children born alive between 1993 and 2012 for whom mother's religion and date of birth were known.<br>**N =** 41,025 children (of whom 25,093 born to Muslim mothers)	4	**A strong association between having been exposed to Ramadan in utero and under-5 mortality was found.**<br>Under-5 mortality rates for children born to Muslim mothers who experienced Ramadan in utero compared to children of non-Muslim mothers during the same period:<br>- Total (not considering Ramadan): HR = 1.15 (95%CI 1.07, 1.24; $p < 0.001$)<br>- Certainly not exposed: HR = 0.97 (95%CI 0.81, 1.17; p = 0.74)<br>- Probably not exposed; HR = 1.11 (95%CI 0.86, 1.43; p = 0.43)<br>- Conceived: HR = 1.33 (95%CI 1.06, 1.66; p = 0.01)<br>- Trimester 1: HR = 1.29 (95%CI 1.12, 1.49; $p < 0.001$)<br>- Trimester 2, HR = 1.22 (95%CI 1.06, 1.40; p = 0.007)<br>- Trimester 3, HR = 1.07 (95%CI 0.91, 1.26; p = 0.39)<br>Born during Ramadan, HR = 1.00 (95%CI 0.82, 1.21; p = 0.98)
Seckin et al. (2014) – Turkey	Prospective cohort study	**Population:** Women with singleton pregnancies of 29 weeks or more of gestation who were fasting for at least 20 days (control group matched for certain characteristics)<br>**N** = 169 (fasted: 82, control: 87)	7	**Fetal development and neonatal outcomes were not significant.**<br>At the end of Ramadan, the changes in BPD, AC, and FL, and FWG were similar between the groups. However, there was a significant association between fasting and decreased AFI.<br>There were no differences between the two groups in birth weight, gestational age at time of delivery, and type of delivery.<br>ΔBPD – fasted: 9.17±1.5mm, control: 9.41±1.3mm (p = 0.26)<br>ΔFL – fasted: 8.62±1.79mm, control: 8.60±1.85mm (p = 0.18)<br>ΔAC – fasted: 41.7±6.7mm, control: 41.3±5.2mm (p = 0.45)<br>ΔAFI – fasted: 20.1±11.2, control: 11.5±6.4 ($p < 0.001$)<br>FWG – fasted: 485.2±162g, control: 461.8±84g (p = 0.16)<br>Birth weight – fasted: 3089±300g, control: 3450±352g (p = 0.09)<br>Gestational age at delivery – fasted: 37.2 weeks, control: 28.6 weeks (p = 0.11)<br>Cesarean delivery – fasted: 24.3%, non-fasted: 25.2% (p = 0.92)<br>UA Doppler, S/D 2.45 ± 0.37 2.41 ± 0.35 0.86<br>MCA Doppler, S/D 4.71 ± 0.72 4.56 ± 0.8 0.98<br>MCA/UA ratio, S/D 1.95 ± 0.43 1.92 ± 0.42 0.72
Shahgheibi et al. (2005) – Iran	Retrospective cohort study	**Population:** Healthy pregnant women aged 25-35 whose third trimester coincided with Ramadan<br>**N =** 163 (fasted: 63, non-fasted:100)	5	**Fasting of pregnant mothers during their third trimester of pregnancy did not have an effect on the growth indices of their newborn babies.**<br>Birth weight – fasted: 3312.90±533.08, non-fasted: 3346.35±377.21 (p = 0.640)<br>Birth length – fasted: 49.74±1.84cm, non-fasted: 49.90cm±1.89 (p = 0.599)<br>Birth head circumference – fasted: 34.65cm±1.57, non-fasted: 34.57cm± 1.37(p = 0.707)<br>LBW – RR in fasted group was 1.9 (0.61-5.98)

Tith et al. (2019) - Canada	Retrospective cohort study	**Population:** data from birth certificates from 1981 to 2017.<br>**N =** 3,123,508 deliveries (among which 78,109 births of Arabic-speaking women).	4	**Among Arabic speakers, Ramadan fasting was associated with the risk of very (28-31) preterm birth:**<br>- Ramadan between weeks 15–21: RR = 1.33 (95% CI 1.06, 1.68)<br>- Ramadan between weeks 22-27: RR = 1.53 (95% CI 1.21, 1.93)<br>Among Arabic speakers, Ramadan fasting was not associated with extreme (22-27 weeks) or late (32-36 weeks) preterm birth regardless of the trimester of pregnancy.<br>Among French or English speakers, Ramadan was not associated with preterm birth.
Van Ewijk (2011) – Indonesia	Retrospective cohort study	**Population:** Sample from Indonesian Family Life Survey, a longitudinal survey. Calculations were made whether a person had been prenatally exposed to Ramadan or not<br>**N** = 29695 (sub-samples used for different analyses)	5	**Exposure to Ramadan during pregnancy may cause negative health effects on the health of offspring, regardless of the stage of pregnancy in which Ramadan took place.**<br>**In fact, some effects only show up, or are stronger when offspring gets older: poorer general health, symptoms for CHD and diabetes, anemia, higher pulse pressure, etc.**<br><br>General health – Muslims: -0.061 ($p < 0.01$), non-Muslims: 0.014 (p = NS)<br>General health – Young Muslims: -0.048 ($p < 0.05$), Young non-Muslims: 0.005 (p = NS)<br>General health – Old Muslims: -0.188 ($p < 0.01$), Old non-Muslims: 0.081 (p = NS)<br>Exposed Muslims ≥50 years – chest pain: 0.088 ($p < 0.10$), wound heal: 0.047 ($p < 0.01$).<br>Exposed Muslims <45 years – anemia: -0.010 (p = NS), high blood pressure: 0.012 (p = NS), pulse pressure: 0.939 ($p < 0.01$)

Van Ewijk, Painter and Roseboom (2013) – Indonesia	Retrospective cohort study	**Population:** Sample from Indonesian Family Life Survey, a longitudinal survey. Women whose date of birth and anthropometric measures were known<br>**N** = 14120 (non-Muslims: 1220 → Not in utero: 143, in utero: 1077, conceived: 110, early: 311, mid: 336, late: 220, born: 100; Muslims: 12900 → not in utero: 1520, in utero: 11380, conceived: 1237, early: 3598, mid: 3272, late: 2196, born: 1076)	5	**Exposure to Ramadan during pregnancy may have lasting consequences for adult body size of the offspring.**<br>Adult Muslims who had been in utero during Ramadan were slightly thinner (adjusted adult BMI: -0.32, 95% CI: -0.57, -0.06) and lighter (NS), and shorter (NS) than those who were not exposed.<br>Those who were conceived during Ramadan also had a smaller stature, being on overage 0.80cm shorter than those who were not exposed to Ramadan prenatally.<br>Among non-Muslims, no such associations were found.<br><br>BMI<br>Not in utero: Reference<br>Conceived during Ramadan: -0.37 (95% CI -0.71, -0.03; $p < 0.05$)<br>Ramadan in early gestation: -0.24 (95% CI -0.53, 0.04; p = NS)<br>Ramadan in mid gestation: -0.42 (95% CI -0.72, -0.13; $p < 0.01$)<br>Ramadan in late gestation: -0.43 (95% CI -0.74, -0.12; $p <0.01$)<br>Born during Ramadan: -0.04 (95% CI -0.40, 0.32; p = NS)<br><br>Weight<br>Not in utero: Reference<br>Conceived during Ramadan: -0.80 (95% CI -1.33, -0.26; $p < 0.01$)
Ziaee et al. (2010) – Iran	Retrospective cohort study	**Population:** All pregnant women aged 20-35 delivering in a referral hospital and fasting during one of the pregnancy trimesters were included<br>**N** = 189 (non-fasted: 66, fasted 1-10 days A: 28, fasted 11-20 days B: 35, fasted > 20 days C: 60 & first trimester: 82, second trimester: 56, third trimester: 51)	5	**No significant difference between means of weight, height, and head circumference of infants with number of days fasted.**<br>**No significant difference between pregnancy outcome parameters and fasting at different trimesters.**<br>However, relative risk of low weight birth was 1.5 times in mothers on fasting at first trimester as compared to non-fasting mothers.<br>Weight – non-fasted: 3.01±0.5kg, A: 3.27±0.5kg, B: 3.01±0.3kg, C: 2.96±0.7kg (p = 0.1)<br>Length – non-fasted: 49.7±2.9cm, A: 49.9±1.7cm B: 50.2±2.5cm, C: 49.2±44cm (p = 0.5)<br>Head circumference – non-fasted: 34.4±1.4cm, A: 34.5±1.5cm, B: 34.4±1.6cm, C: 33.7±2.3cm (p = 0.1)<br>Weight – first trimester: 2.998±0.676kg, second trimester: 2.949±0.733kg, third trimester: 3.105±0.585kg (p = 0.3)<br>Length – first trimester: 49.0±3.9cm, second trimester: 50.1±4.8cm, third trimester: 20.2±3.3cm (p = 0.6)<br>Head circumference – first trimester: 33.9±2.1cm, second trimester: 33.9±2.5cm, third trimester: 34.4±1.9cm (p = 0.4)
